# Parvalbumin Interneurons Determine Emotional Valence Through Modulating Accumbal Output Pathways

**DOI:** 10.3389/fnbeh.2019.00110

**Published:** 2019-05-14

**Authors:** Xi Chen, Zhiyuan Liu, Chaonan Ma, Lan Ma, Xing Liu

**Affiliations:** The State Key Laboratory of Medical Neurobiology and MOE Frontiers Center for Brain Science, School of Basic Medical Sciences and the Institutes of Brain Science, Fudan University, Shanghai, China

**Keywords:** parvalbumin interneurons, nucleus accumbens, calretinin interneurons, conditioned place preference, conditioned place aversion

## Abstract

Parvalbumin (PV) expressing GABAergic interneurons provide large source of GABA to spiny projection neurons (SPNs) in the striatum. However, the roles of PV^+^ interneurons in the regulation of SPNs in the ventral striatum and emotional states are largely unknown. Here, we investigated whether stimulation of ventral striatal (accumbal) PV^+^ interneurons would drive emotional valence in mice. We found that during conditioned place preference (CPP) training, activation of accumbal PV^+^ interneurons evoked place preference while suppressing them resulted in conditioned place aversion (CPA). Activation of PV^+^ interneurons during place conditioning increased *Fos* expression in SPNs in the direct pathway (dSPNs) and impaired lithium chloride-induced CPA. Activation of dSPNs and SPNs in the indirect pathway (iSPNs) induced CPP and CPA, respectively; conversely, suppression of dSPNs or iSPNs induced CPA or CPP. In addition, activation or suppression of calretinin-expressing (CR) GABAergic interneurons did not induce place preference or aversion. These data suggest that PV^+^ interneurons can bidirectionally determine the emotional valence through their regulation of accumbal SPN activities and raise the possibility that manipulation of PV^+^ interneuron activity may have the potential to alter emotional valence and treat related mental disorders.

## Introduction

The striatum is the primary input nuclei of the basal ganglia. The ventral striatum, also known as the nucleus accumbens (NAc), plays a critical role in processing motivated behavior and reward-related learning (Floresco, 2015). The NAc receives a wide variety of glutamatergic inputs from cortex, thalamus, amygdala, and hippocampus (Britt et al., 2012; MacAskill et al., 2012, 2014), as well as dopaminergic projections from the ventral tegmental area (VTA; Gerfen and Surmeier, 2011; Pignatelli and Bonci, 2015), and transforms these converging glutamatergic and dopaminergic inputs into two output streams, named the direct and the indirect pathways (Lobo et al., 2010; Bock et al., 2013; Zhang et al., 2015). Although the actions of these output pathways are complicated, a simplified model of the NAc circuit posits that the direct pathway promotes rewarding effects and approaching behavior while the indirect pathway facilitates aversive effects and avoidance (Kravitz et al., 2012; Calipari et al., 2016). Imbalance in the activity of these two pathways is hypothesized to underlie addiction, anhedonia and mental disorders (Robison and Nestler, 2011; Kravitz and Kreitzer, 2012).

GABAergic spiny projection neurons (SPNs) constitute as much as 90% of the NAc neurons (Lobo, 2009). SPNs are classically divided into two approximately equally sized populations based on their projection targets and expression of dopamine receptors. SPNs in direct pathway (dSPNs) mainly express G_s/olf_-coupled dopamine D1 receptors and send dense projections to the VTA. SPNs in indirect pathway (iSPNs) mainly express G_i/o_-coupled dopamine D2 receptors and send axons to the ventral pallidum (VP; Soares-Cunha et al., 2016). The remaining 5%–10% of neurons in NAc are GABAergic interneurons (Tepper et al., 2010; Straub et al., 2016). Three main classes of GABAergic interneurons in the NAc are classically recognized: parvalbumin-positive (PV^+^) fast-spiking interneurons (FSIs), calretinin-expressing (CR^+^) GABAergic interneurons and somatostatin-positive (SST^+^) low-threshold-spiking interneurons (LTSIs; Tepper et al., 2008). Although local GABAergic signaling strongly regulates the activity of both dSPNs and iSPNs in striatum (Nisenbaum and Berger, 1992; Wilson, 2007), the roles of GABAergic interneurons in regulating the NAc output pathways and the behavioral responses to psychostimulants remain poorly understood.

The majority of GABAergic control of striatal SPNs arises through feedforward inhibition from local GABAergic interneurons (Tepper et al., 2004). Of those, most feedforward inhibition is mediated by PV^+^ interneurons (Koos and Tepper, 1999). PV^+^ interneurons show extensive, strong inhibitory synapses onto the somata and proximal dendrites of SPNs, and contribute an important role in organization of the dorsal striatal microcircuit (Straub et al., 2016). Suppression, not activation, of dorsal striatal PV^+^ interneurons inhibited initial expression of sucrose conditioned responses (Lee et al., 2017). Studies suggest that PV^+^ interneurons regulate striatal output and the performance during reward associative learning (Burke et al., 2017). In comparison, the roles of accumbal PV^+^ interneurons in the modulation of accumbal SPNs and the behavioral responses to psychostimulants are less well understood. Some recent pioneering work shows that ventral hippocampus evokes feedforward inhibition on accumbal PV^+^ interneurons (Scudder et al., 2018) and activation of amygdala-NAc shell projections expedites acquisition of cocaine self-administration through a PV-mediated inhibition of SPNs (Yu et al., 2017). A recent work also shows a requirement of PV^+^ interneurons in the NAc in AMPH induced behavioral adaptations (Wang et al., 2018). Thus, PV^+^ interneurons are poised to have a major impact on shaping accumbal outputs. To demonstrate this, we stimulated PV^+^ in the NAc core (NAcc) directly to address the roles of PV^+^ interneurons in the regulation of accumbal output pathways and LiCl-induced place aversion.

## Materials and Methods

### Animals

D1::Cre (#034258-UCD) and D2::Cre (#032108-UCD) mice were purchased from The Mutant Mouse Resource and Research Center. PV::Cre (#012358) and CR::Cre (#010774) mice were purchased from The Jackson Laboratory. Eight- to 10-week-old male mice were used. For all the behavioral experiments, four mice were group housed per cage under a 12:12-h light/dark cycle with food and water provided *ad libitum*. Before behavioral experiments, animals were handled by experimenters for 5 min per day and at least for three consecutive days. All the experiments were conducted in the animals’ light cycle and in accordance with the National Institutes of Health Guide for the Care and Use of Laboratory Animals and were approved by Animal Care and Use Committee of Shanghai Medical College of Fudan University.

### Viral Vectors and Reagents

The AAVs preparation with a titer exceeding 2 × 10^12^ vector genome (vg) ml^−1^ were used for infection. AAV_9_-EF1α-DIO-hM3Dq-mCherry and AAV_9_-EF1α-DIO-hM4Di-mCherry were generated and packaged by Taitool Biological Co., Ltd. AAV_9_-EF1α-DIO-mCherry and AAV_9_-EF1α-DIO-ChR2-mCherry were generated and packaged by BrainVTA Co., Ltd.

Clozapine-N-oxide (CNO, #C0832, Sigma) was dissolved in saline at 0.2 mg/ml and injected intraperitoneally (1 mg/kg, i.p.) 30 min prior to conditioning. Lithium chloride (LiCl, #746460, Sigma) was dissolved in saline at 20 mg/ml and administrated at a dose of 100 mg/kg (i.p.) for mouse LiCl-conditioned place aversion (CPA) model.

### Stereotaxic Virus Injection and Optical Cannula Implantation

For virus injection, 6–8-weeks-old mice were anesthetized with isoflurane (3.5% induction, 1.5%-2% maintenance), placed in a stereotaxic apparatus (Stoelting Instruments, Wood Dale, IL, USA), and injected with small amounts of AAV (200 nl) into the NAcc. The virus was delivered using a 10 μl syringe and a 36 gauge blunt needle under the control of a UMP3 ultra micro pump (World Precision Instruments, Sarasota, FL, USA) with a controlled volume and flow rate (200 nl at 50 nl/min). The virus was injected into both sides of the NAcc [anteroposterior (AP): +1.7 mm; mediolateral (ML): ±1.2 mm; dorsoventral (DV): –4.3 mm from bregma]. After injection, the needle was left for an additional 10 min and was then slowly removed. A small craniotomy was performed and two or three holes were drilled. For chemogenetic stimulation, two holes were drilled for bilateral virus injection in NAcc. For optogenetic stimulation, one hole was drilled for screw implantation. The other two holes were drilled for the virus injection and optical fiber (Anilab, China) implantation in the virus injection sites. All behavioral experiments were conducted at least 2 weeks after virus injection.

### Behavioral Tests With Optogenetic and Chemogenetic Manipulations of NAcc PV^+^ Interneurons Activity

The unbiased conditioned place preference (CPP)/A paradigm was applied as described previously (Liu et al., 2015). The CPP/A apparatus consisted of two compartments (15 × 15 × 20 cm^3^) with different patterned flooring and walls separated by a removable board to allow the mice to discriminate them. One compartment had black and white striped walls and frosted floor with a black ceiling. The other compartment had black and white checkered walls and black floor with white ceiling.

#### Pre-test Session

One day before the conditioning, the mice with or without bilateral optical fibers were released from middle of the conditioning apparatus and allowed to explore the full extent of the apparatus freely for 15 min to test their innate compartment preference. Mice that spent >65% ( >585 s) or <35% (<315 s) of the total time (900 s) in one side were eliminated from subsequent CPP experiments.

#### CPP Training Session

We use a removable board to divide the apparatus into two compartments. Mice were presented with two conditioning trials for 30 min separated by 6 h each day. For optogenetic stimulation, the mice were confined in one of the conditioning compartments for 30 min with optical stimulation (473 nm, 20 Hz frequency, 1 burst of 10 pulses, 15 ms pulse width, every 10 s, 10 mW) in the morning and confined in the other compartment for 30 min without optical stimulation in the afternoon. For chemogenetic stimulation, the mice were confined in one compartment for 30 min with CNO treatment (1 mg/kg i.p. 30 min before the conditioning) in the morning and confined in the other compartment for 30 min with saline treatment (4 ml/kg i.p. 30 min before the conditioning). The laser/CNO and no laser/Saline paired conditioning was performed alternatively in the morning or afternoon and repeated for 3 days.

#### Test Session

One day after conditioning, the board was removed and the mice were allowed for exploring the entire CPP/A apparatus for 15 min to assess their preference or avoidance for the conditioning compartments. The body position of the mice during the Pre-test and Test sessions was tracked by the EthoVision XT video tracking software (Noldus Information Technology, Wageningen, Netherlands). The preference or avoidance score was determined as time (s) spent in optical stimulation paired side minus non-optical stimulation paired side or CNO paired side minus saline paired side.

#### LiCl-CPA

In the Pre-test session, the mice were allowed to explore the CPA apparatus for 15 min and test their innate compartment preference. In the CPA training session, the mice were presented with two conditioning trials for 30 min separated by 6 h for 3 days: one compartment with LiCl treatment (100 mg/kg, i.p.) and optical stimulation (473 nm, 20 Hz frequency, 1 burst of 10 pulses, 15 ms pulse width, every 10 s, 10 mW) and the opposite compartment with saline treatment and no optical stimulation. In the Test session, the mice were placed in CPA apparatus and allowed free exploration of two compartments 1 day after training session. Their avoidance for the conditioning compartment was assessed.

### High-Resolution Fluorescence *in situ* Hybridization by RNAscope (FISH by RNAscope)

Thirty minutes after optogenetic stimulation, the mice were perfused intracardiacally with saline first, then with 4% paraformaldehyde in 0.1 M Na_2_HPO_4_/NaH_2_PO_4_ buffer (pH = 7.5) and the brains were removed. After post-fixation in 4% paraformaldehyde for 4 h, the samples were stored in 30% sucrose/PBS for 3 days. FISH was performed on the fixed frozen brain slices with 10 μm thick, following the RNAscope procedures (Advanced Cell Diagnostics, Inc., Newark, CA, USA). In brief, frozen sections (10 μm thick) were cut coronally through the NAc formation. Sections were thaw-mounted onto Superfrost Plus Microscope Slides (Fisher Scientific, Waltham, MA, USA) and pretreated for protease digestion for 10 min at room temperature. Sections were then incubated with probes of mouse *Fos*, *Drd1* and *Drd2* (*Fos*, accession No: NM_010234.2, target region 407–1427; *Drd1*, accession No: NM_010076.3, target region 444–1358; *Drd2*, accession No: NM_010077.2, target region 69–1175) for 2 h at 40°C with labeled probe mixture per slide. The nonspecifically hybridized probe was removed by washing the sections in 1× washing buffer at room temperature, followed by Amplifier 1-FL for 30 min, Amplifier 2-FL for 30 min and Amplifier 3-FL for 15 min at 40°C. Each amplifier was removed by washing with 1× washing buffer for 2 min at room temperature. The slides were incubated with HRP-C1, HRP-C2, and HRP-C3 followed by TSA-fluorophore each. The slides were viewed, analyzed, and photographed with an LSM 510 microscope (Zeiss). At least three independent experiments have been performed and imaged from five male mice in each group.

### Statistical Analysis

Experimental data were presented as the mean ± standard error of the mean (SEM) and analyzed by Sigma plot 12.5. Data of immunofluorescence were analyzed by two-way Analysis of Variance (ANOVA). Data from behavioral tests were analyzed by two-way ANOVA with repeated measures followed by the *Bonferroni’s post hoc* test with sessions as a within-subjects factor and AAV as a between-subjects factor. *P* < 0.05 was considered statistically significant, *p or ^#^*p* < 0.05, **p or ^##^*p* < 0.01, ***p or ^###^*p* < 0.001.

## Results

### Stimulation of ChR2 in NAcc PV^+^ Interneurons Evokes Place Preference

**Figure 1:**
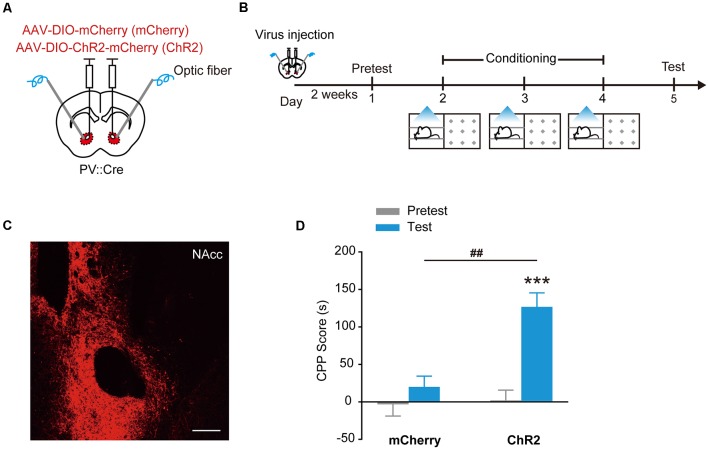
Optogenetic activation of ChR2 in accumbal Parvalbumin-positive (PV^+^) interneurons evoked conditioned place preference (CPP). **(A,B)** Virus injection and behavioral scheme. AAV_9_-EF1α-DIO-mCherry (mCherry) or AAV_9_-EF1α-DIO-ChR2-mCherry (ChR2) was infused into the NAcc of PV::Cre mice, and optical fiber was implanted in the nucleus accumbens core (NAcc). The mice were presented with daily conditioning trials for 3 days: one compartment paired with laser stimulation and the other compartment without laser stimulation. **(C)** Representative image of ChR2-mCherry expression in NAcc of PV::Cre mice. Scale bar: 200 μm. **(D)** Optogenetic activation of ChR2 in NAcc PV^+^ interneurons promoted CPP formation. mCherry, *n* = 20; ChR2, *n* = 10, ***p < 0.001 vs. Pretest group, ^##^*p* < 0.01 vs. mCherry group. Data are expressed as mean ± standard error of the mean (SEM).

Channelrhodopsin-2 (ChR2) is a cation channel that is originally identified in green algae. Stimulation of ChR2 with blue light causes depolarization and the firing of action potentials in the stimulated cells (Boyden et al., 2005), demonstrated as a powerful experimental tool to activate specific neural circuits and behaviors (Bernstein and Boyden, 2011; Tye and Deisseroth, 2012). To study the roles of PV^+^ interneurons in the NAcc in the regulation of motivational behaviors, we used optogenetics to manipulate the activity of PV^+^ interneurons. PV::Cre mice injected with AAV_9_-EF1α-DIO-mCherry or AAV_9_-EF1α-DIO-ChR2-mCherry in the NAcc were placed in one compartment of CPP apparatus, and received optical stimulation through optical fibers placed over the NAcc to activate ChR2 in PV^+^ interneurons during place conditioning (Figures 1A–C). During subsequent CPP test, NAcc^PV^:ChR2 mice showed significant place preference as they spent more time in the stimulation-paired compartment than NAcc^PV^: mCherry mice (control mice) did (Figure 1D, *F*_treatment × session (1,28)_ = 10.244, *p* = 0.003, two-way RM ANOVA). These data showed that optogenetic activation of ChR2 in accumbal PV^+^ interneurons induced CPP, suggesting activation of these GABAergic interneurons in the NAcc supports rewarding and approaching behavior.

### Stimulation of hM3Dq or hM4Di in NAcc PV^+^ Interneurons Induces Place Preference or Avoidance

**Figure 2:**
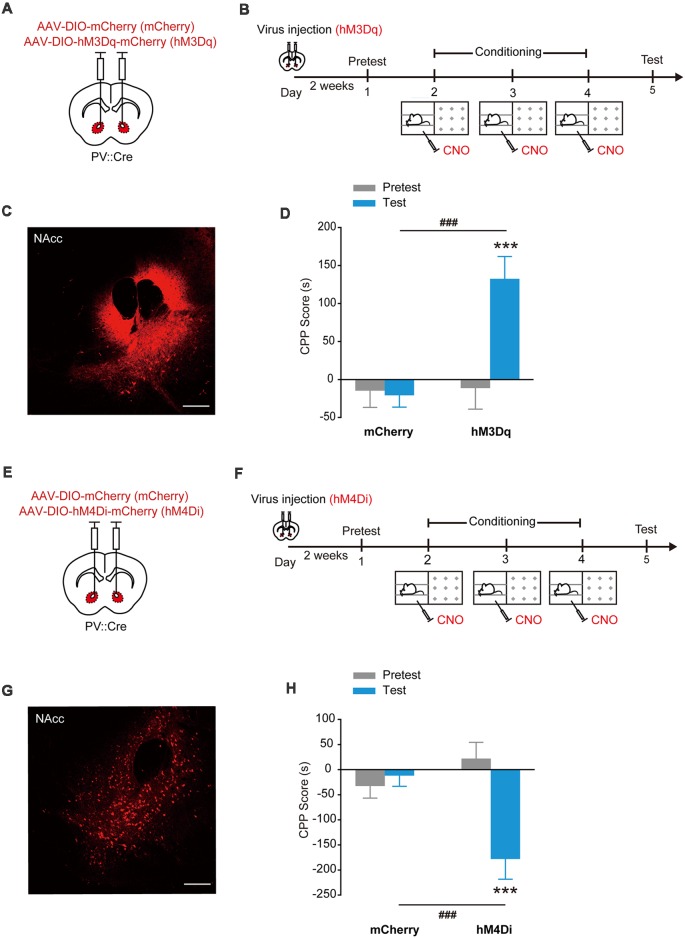
Chemogenetic activation of hM3Dq or hM4Di in accumbal PV^+^ interneurons induced CPP or conditioned place aversion (CPA). **(A,B)** Virus injection and behavioral scheme. AAV_9_-EF1α-DIO-mCherry (mCherry) or AAV_9_-EF1α-DIO-hM3Dq-mCherry (hM3Dq) was infused into the NAcc of PV::Cre mice. The mice were presented with daily conditioning trials for 3 days: one compartment paired with Clozapine-N-oxide (CNO) treatment and the other compartment with saline injection. **(C)** Expression of hM3Dq-mCherry in NAcc of PV::Cre mice. Scale bar: 200 μm. **(D)** Chemogenetic activation of NAcc PV^+^ interneurons promoted CPP formation. mCherry, *n* = 22; hM3Dq, *n* = 13, ***p < 0.001 vs. Pretest group, ^###^*p* < 0.001 vs. mCherry group. **(E,F)** Virus injection and behavioral scheme. AAV_9_-EF1α-DIO-mCherry (mCherry) or AAV_9_-EF1α-DIO-hM4Di-mCherry (hM4Di) was infused into the NAcc of PV::Cre mice. The mice were presented with daily conditioning trials for 3 days: one compartment with CNO treatment and the other compartment with saline injection. **(G)** Expression of hM4Di-mCherry in NAcc of PV::Cre mice. Scale bar: 200 μm. **(H)** Chemogenetic inhibition of NAcc PV^+^ interneurons promoted CPA formation. mCherry, *n* = 16; hM4Di, *n* = 16, ***p < 0.001 vs. Pretest group, ^###^*p* < 0.001 vs. mCherry group. Data are expressed as mean ± SEM.

The designer receptors exclusively activated by designer drugs (DREADDs) are another powerful tools to manipulate cell activities (Dong et al., 2010). The human M3 and M4 muscarinic receptors (hM3Dq and hM4Di) are strongly activated in response to a synthetic ligand, CNO, inducing an enhancement of neuronal excitability and suppression of neuronal firing, respectively (Dong et al., 2010; Atasoy et al., 2012; López et al., 2016; Wei et al., 2018). To further confirm that optogenetic activation of NAcc PV^+^ interneurons can evoke place preference, we injected AAV_9_-EF1α-DIO-hM3Dq-mCherry, AAV_9_-EF1α-DIO-hM4Di-mCherry or AAV_9_-EF1α-DIO-mCherry into the NAcc of PV::Cre mice and chemogenetically activated hM3Dq or hM4Di in NAcc PV^+^ interneurons with CNO (Figures 2A–C). Similarly, these mice were placed in one compartment paired with a Clozapine-N-oxide (CNO) injection and the other compartment with a saline injection. Consistently, in the subsequent test, the NAcc^PV^:hM3Dq mice spent significantly more time in CNO-paired compartment and developed CPP, while control mice did not develop CPP (Figure 2D, *F*_treatment × session (1,33)_ = 13.816, *p* < 0.001, two-way RM ANOVA). Conversely, the NAcc^PV^:hM4Di mice spent less time in CNO-paired compartment and developed CPA (Figures 2E–H, *F*_treatment × session (1,30)_ = 13.923, *p* < 0.001, two-way RM ANOVA). These data showed that activation of hM3Dq in accumbal PV^+^ interneurons promoted CPP formation and activation of hM4Di in accumbal PV^+^ interneurons promoted CPA formation, thus indicating that activation and suppression of PV^+^ GABAergic interneurons in the NAcc promotes opposite emotional valence.

### Stimulation of ChR2 in NAcc PV^+^ Interneurons Induces Activation of D1^+^ SPN Population

**Figure 3:**
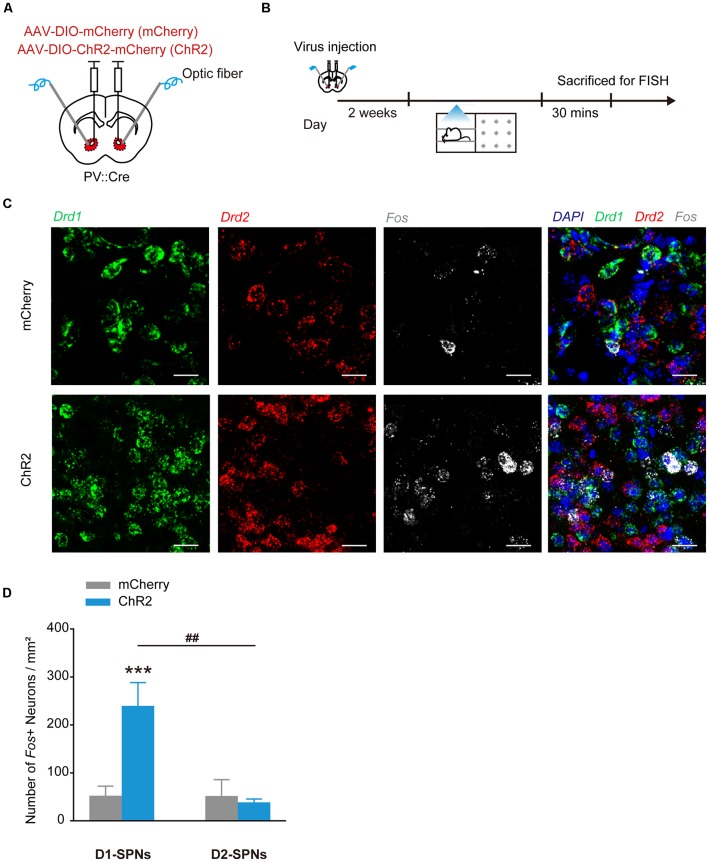
Optogenetic activation of ChR2 in accumbal PV^+^ interneurons increased *Fos* mRNA expression in dSPNs in the NAcc. **(A,B)** Virus injection and behavioral scheme. AAV_9_-EF1α-DIO-mCherry (mCherry) or AAV_9_-EF1α-DIO-ChR2-mCherry (ChR2) was infused into the NAcc of PV::Cre mice, and optical fiber was implanted in the NAcc. Two to three weeks after surgery, the mice were presented with laser stimulation and sacrificed for FISH 30 min after laser stimulation. **(C)** Representative image of FISH for *Drd1* mRNA (green), *Drd2* mRNA (red), *Fos* mRNA (white) expression in the NAcc of PV::Cre mice. Scale bar: 20 μm. **(D)** Cell counts and percentage of *Fos*^+^/*Drd1*^+^ and *Fos*^+^/*Drd2*^+^ cells. mCherry, *n* = 5; ChR2, *n* = 5, ***p < 0.001 vs. mCherry group, ^##^*p* < 0.01 vs. D2-spiny projection neurons (SPNs). Data are expressed as mean ± SEM.

GABAergic interneurons provide strong synapses on SPNs in the NAcc, thus the reward or aversion evoked by the stimulation of PV^+^ interneurons may be caused by their collateral transmission on SPNs. To determine the potential regulation of PV^+^ interneurons on SPNs in direct and indirect pathways, we injected AAV_9_-EF1α-DIO-ChR2-mCherry into the NAcc of PV::Cre mice and tested the *Fos* expression levels in D1^+^ and D2^+^ SPNs by *in situ* hybridization after optogenetic stimulation of PV^+^ interneurons (Figures 3A,B). In NAcc^PV^:ChR2 mice, the counts of *Fos*^+^/*Drd1*^+^ neurons were greatly increased and the proportion of *Fos*^+^/*Drd1*^+^ neurons was significantly higher than proportion of *Fos*^+^/*Drd2*^+^ neurons in the NAcc after optogenetic stimulation (Figures 3C,D, *F*_(1,16)_ = 10.088, *p* = 0.006, two-way ANOVA).

### Stimulation of hM3Dq or hM4Di in NAcc dSPNs and iSPNs Evokes Opposing Emotional Valence

**Figure 4:**
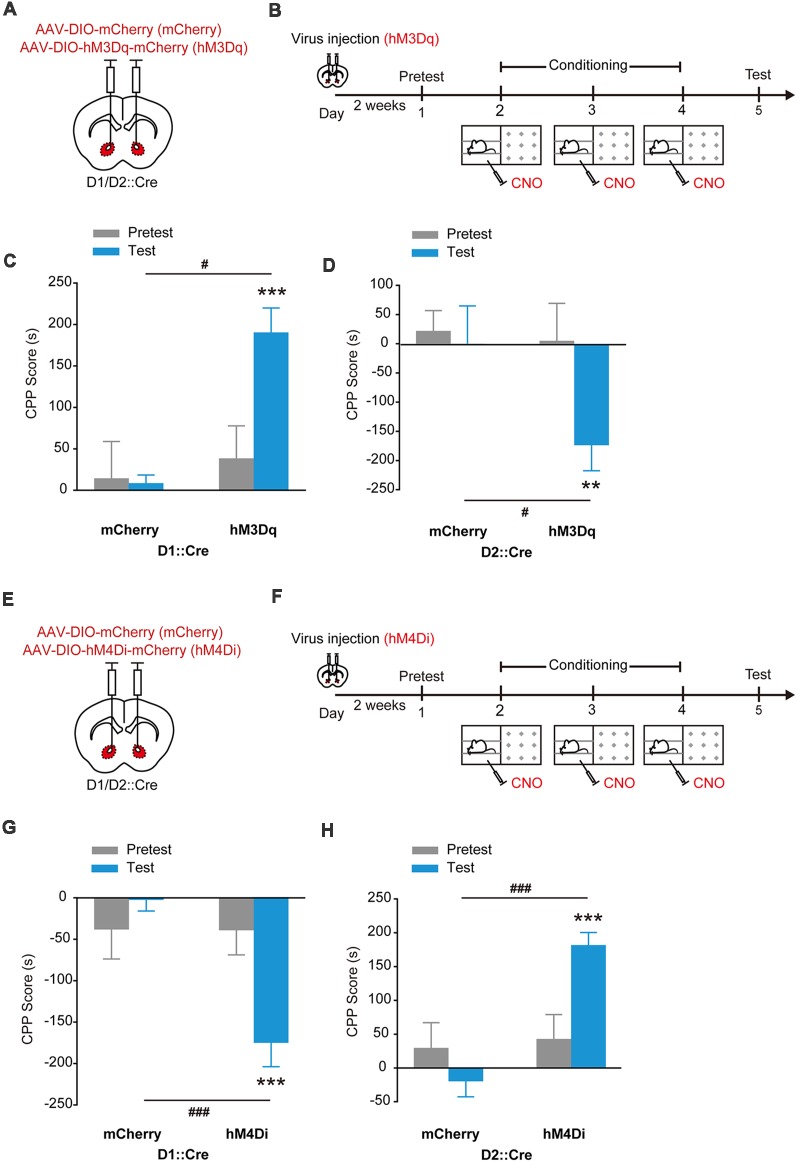
Chemogenetic activation of hM3Dq or hM4Di in accumbal dSPNs and iSPNs induced opposing emotional valence. **(A,B)** Virus injection and behavioral scheme. AAV_9_-EF1α-DIO-mCherry (mCherry) or AAV_9_-EF1α-DIO-hM3Dq-mCherry (hM3Dq) was infused into the NAcc of D1::Cre and D2::Cre mice. Two to three weeks after surgery, the mice were presented with daily conditioning trials for 3 days: one compartment paired with CNO treatment and the other compartment paired with saline injection. **(C,D)** Chemogenetic activation of dSPNs promoted CPP formation and activation of iSPNs promoted CPA formation. mCherry, *n* = 8; D1::Cre, *n* = 10; D2::Cre, *n* = 8, ***p < 0.001 vs. Pretest group, ^#^*p* < 0.05 vs. mCherry group of D1::Cre mice; **p < 0.01 vs. Pretest group, ^#^*p* < 0.05 vs. mCherry group of D2::Cre mice. **(E,F)** Virus injection and behavioral scheme. AAV_9_-EF1α-DIO-mCherry (mCherry) or AAV_9_-EF1α-DIO-hM4Di-mCherry (hM4Di) was infused into the NAcc of D1::Cre and D2::Cre mice. Two to three weeks after surgery, the mice were presented with daily conditioning trials for 3 days: one compartment paired with CNO treatment and the other compartment paired with saline injection. **(G,H)** Chemogenetic inhibition of dSPNs promoted CPA formation and inhibition of iSPNs promoted CPP formation. mCherry, *n* = 11; D1::Cre, *n* = 11; D2::Cre, *n* = 12, ***p < 0.001 vs. Pretest group, ^###^*p* < 0.001 vs. mCherry group. Data are expressed as mean ± SEM.

As the main output of NAcc, the effects of stimulation of dSPNs and iSPNs on reward or aversion were investigated. We injected AAV_9_-EF1α-DIO-hM3Dq-mCherry or AAV_9_-EF1α-DIO-hM4Di-mCherry into the NAcc of D1::Cre mice and applied CNO when the mice were placed in the paired compartment (Figures 4A,B,E,F). The NAcc^D1^:hM3Dq mice spent significantly more time in CNO-paired compartment (Figure 4C, *F*_treatment × session (1,16)_ = 7.963, *p* = 0.012, two-way RM ANOVA) and the NAcc^D1^:hM4Di mice spent significantly less time in CNO-paired compartment (Figure 4G, *F*_treatment × session (1,20)_ = 15.841, *p* < 0.001, two-way RM ANOVA). Next, we injected AAV_9_-EF1α-DIO-hM3Dq-mCherry or AAV_9_-EF1α-DIO-hM4Di-mCherry into the NAcc of D2::Cre mice and applied chemogenetical stimulation (Figures 4A,B,E,F). Conversely, the NAcc^D2^:hM3Dq mice developed CPA (Figure 4D, *F*_treatment × session (1,14)_ = 6.112, *p* = 0.027, two-way RM ANOVA) and the NAcc^D2^:hM4Di mice developed CPP (Figure 4H, *F*_treatment × session (1,21)_ = 14.650, *p* < 0.001, two-way RM ANOVA). These data showed that D1-SPN activation and D2-SPN deactivation both induced place preference, while D1-SPN deactivation and D2-SPN activation resulted in placed avoidance, suggesting that stimulation of NAcc dSPNs and iSPNs promotes opposing emotional valence.

### Stimulation of ChR2 in NAcc PV^+^ Interneurons Impairs LiCl-Induced Place Aversion

**Figure 5:**
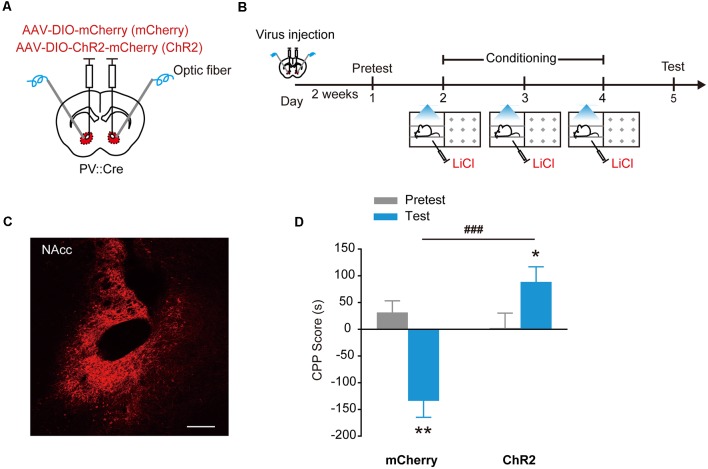
Optogenetic activation of ChR2 in accumbal PV^+^ interneurons impaired LiCl-induced CPA. **(A,B)** Virus injection and behavioral scheme. AAV_9_-EF1α-DIO-mCherry (mCherry) or AAV_9_-EF1α-DIO-ChR2-mCherry (ChR2) was infused into the NAcc of PV::Cre mice, and optical fiber was implanted in the NAcc. Two to three weeks after surgery, the mice were presented with daily conditioning trials for 3 days: one compartment paired with LiCl treatment (100 mg/kg) and laser stimulation and the other compartment paired with saline. **(C)** Expression of ChR2-mCherry in NAcc of PV::Cre mice. Scale bar: 200 μm. **(D)** Summary bar graphs of CPP scores. mCherry, *n* = 12; ChR2, *n* = 12, **p < 0.01 vs. Pretest mCherry group, *p < 0.05 vs. Pretest ChR2 group, ^###^*p* < 0.001 vs. mCherry group. Data are expressed as mean ± SEM.

To test the effects of activation of PV^+^ interneurons on LiCl-induced place aversion, we injected AAV_9_-EF1α-DIO-ChR2-mCherry into the NAcc of PV::Cre mice and locally optogenetically activated ChR2 expressed in PV^+^ interneurons during LiCl conditioning (Figures 5A–C). PV^+^ interneuron activation significantly increased the duration mice spent in LiCl-paired side (Figure 5D, *F*_treatment × session (1,22)_ = 28.986, *p* < 0.001, two-way RM ANOVA). These data showed that activation of ChR2 in NAcc PV^+^ interneurons impaired CPA induced by LiCl.

### Stimulation of hM3Dq or hM4Di in NAcc CR^+^ Interneurons Does Not Induce Place Preference or Aversion

**Figure 6:**
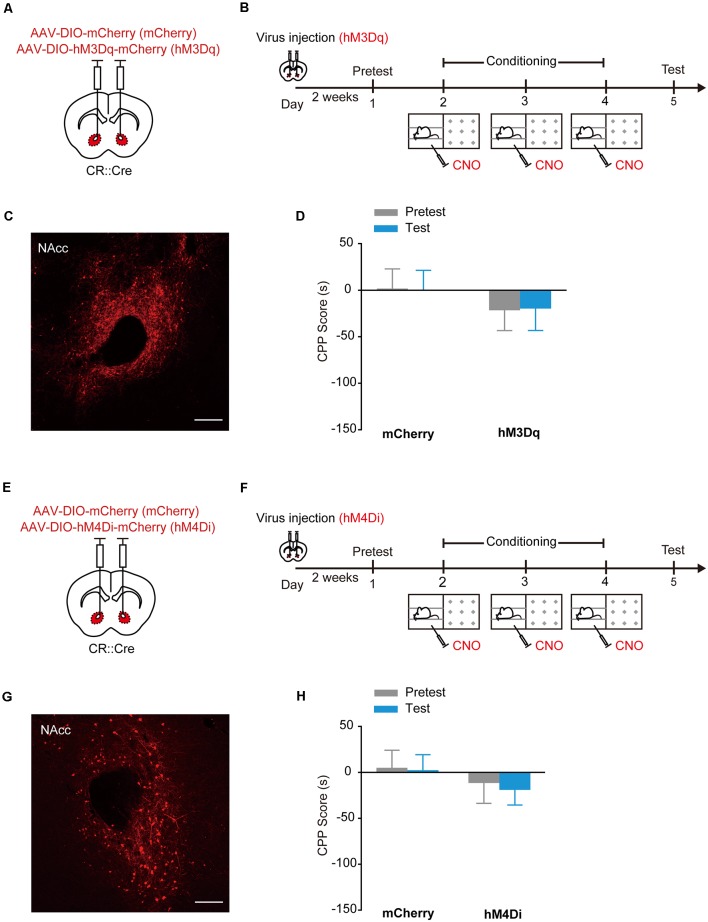
Chemogenetic activation of hM3Dq or hM4Di in accumbal CR^+^ interneurons did not induce CPP or CPA. **(A,B)** Virus injection and behavioral scheme. AAV_9_-EF1α-DIO-mCherry (mCherry) or AAV_9_-EF1α-DIO-hM3Dq-mCherry (hM3Dq) was infused into the NAcc of CR::Cre mice. Two to three weeks after surgery, the mice were presented with daily conditioning trials for 3 days: one compartment paired with CNO treatment and the other compartment paired with saline injection. **(C)** Expression of hM3Dq-mCherry in NAcc of CR::Cre mice. Scale bar: 200 μm. **(D)** Summary bar graphs of CPP scores. mCherry, *n* = 18; hM3Dq, *n* = 12. **(E,F)** Virus injection and behavioral scheme. AAV_9_-EF1α-DIO-mCherry (mCherry) or AAV_9_-EF1α-DIO-hM4Di-mCherry (hM4Di) was infused into the NAcc of CR::Cre mice. Two to three weeks after surgery, the mice were presented with conditioning trials: one compartment with CNO treatment and the other compartment with saline injection. After 3-day conditioning, the CPP test was performed. **(G)** Expression of hM4Di-mCherry in NAcc of CR::Cre mice. Scale bar: 200 μm. **(H)** Summary bar graphs of CPP scores. mCherry, *n* = 21; hM4Di, *n* = 17. Data are expressed as mean ± SEM.

To test whether activation of CR^+^ interneurons in the NAcc can drive preference as PV^+^ interneurons do, we injected AAV_9_-EF1α-DIO-hM3Dq-mCherry (Figures 6A–C) or AAV_9_-EF1α-DIO-hM4Di-mCherry (Figures 6E–G) into the NAcc of CR::Cre mice and conditioned the mice with CNO. The NAcc^CR^:hM3Dq and NAcc^CR^:hM4Di mice did not show preference or avoidance for CNO-paired compartment (Figures 6D,H, hM3Dq: *F*_treatment × session (1,28)_ = 0.006, *p* = 0.939; hM4Di: *F*_treatment × session (1,36)_ = 0.027, *p* = 0.871, two-way RM ANOVA). These data suggest that stimulation of NAcc CR^+^ interneuron may not induce reward or aversion. Taken together, these data indicate that PV^+^ and CR^+^ interneurons in the NAcc might have distinct roles in driving emotional valence.

## Discussion

Although the feedforward inhibition mediated by PV^+^ interneurons has long been recognized as a key circuit mechanism in regulating the functional output of principal neurons in the striatum, prefrontal cortex, amygdala and other brain regions, the PV regulation remains unclear in the NAc, a brain region critically implicated in psychological diseases. Using PV::Cre mice combined with optogenetic and chemogenetic tools, our current study characterized that activation of PV^+^ interneurons drove CPP and suppression of PV^+^ interneurons drove CPA. Stimulation of PV^+^ interneurons induced dSPN activation and impaired LiCl-induced CPA. Thus, PV^+^ interneurons can bidirectionally determine the emotional valence by regulation of accumbal output pathways.

A pioneering work suggests that optogenetically suppressing or activating striatal PV^+^ interneurons reduces SPN activity and disrupts reward anticipatory behavior (Lee et al., 2017). However, our study indicates that the regulation of accumbal PV^+^ interneurons is different from the unidirectional control of striatal PV^+^ interneurons. We found that activation or suppression of PV^+^ interneurons drove CPP or CPA, suggesting stimulating PV^+^ interneurons can evoke opposing emotional states, which might be attributed to PV bidirectional regulation of accumbal SPN activities. Another work suggests that activation of PV^+^ interneurons in the NAc shell by stimulating amygdala-NAc shell glutamatergic projection expedites cocaine self-administration (Yu et al., 2017), suggesting that activation of PV^+^ interneurons in the NAc shell promotes rewarding response to cocaine. One recent work shows that silencing accumbal PV^+^ interneurons selectively inhibited locomotor sensitization by repeated AMPH treatment and blocked AMPH-induced CPP (Wang et al., 2018), suggesting that inhibition of PV^+^ neurons impairs rewarding responses to AMPH. These researches suggest PV^+^ interneurons might bidirectionally regulate accumbal output pathways and responses to psychoactive substances, consistent with our optogenetic and chemogenetic stimulation data. Thus, the functions of PV^+^ interneurons are distinct in dorsal and ventral striatum.

PV^+^ interneurons are sporadically distributed throughout the central nerves system and each PV^+^ interneuron innervates tens to hundreds of principal neurons (Hu et al., 2014). Striatal PV^+^ interneurons are monosynaptic coupling with SPNs (Planert et al., 2010; Tepper et al., 2010). In the dorsal striatum, PV^+^ interneurons robustly connect to dSPNs and iSPNs and mediate the bulk of feedforward inhibition. In addition, PV^+^ interneurons preferentially target dSPNs over iSPNs, suggesting a potential mechanism for rapid pathway-specific regulation of striatal output pathways (Gittis et al., 2010). In the ventral striatum, optogenetic activation of PV^+^ interneurons directly inhibits D1^+^ and D1^−^ SPNs (Scudder et al., 2018), but it is not clear whether accumbal PV^+^ interneurons preferentially target dSPNs or iSPNs. In this study, we found that activation of PV^+^ interneurons significantly increased *Fos* mRNA expression level preferentially in the D1^+^ SPNs, suggesting that activation of PV^+^ interneurons in the NAcc promotes place preference likely through its disinhibition of D1-SPNs. These data showed that accumbal PV^+^ interneurons might preferentially target iSPNs over dSPNs. One possibility is that optical stimulation of PV^+^ interneurons inhibits iSPN collateral transmission to dSPNs and finally disinhibits the dSPNs. The second possibility is that 20 Hz optogenetic stimulation of PV^+^ interneurons may result in synchronization of SPN firing *via* feedforward inhibition and thus supports D1^+^ SPNs reward processing. The third possibility is that the accumbal PV^+^ interneurons might have disynaptic inhibitory coupling through intermediary interneurons, such as NPY^+^, or other PV^+^ interneurons, which directly regulate dSPNs (Burke et al., 2017). It is possible that other interneurons, like NPY^+^ neurons, may provide even stronger inhibition of SPNs than PV^+^ interneurons (Ibáñez-Sandoval et al., 2011). Thus, stimulating a strong inhibitory circuit does not necessarily result in disinhibition. The direct evidence needs further investigation by electrophysiological experiments.

CPA is generally used to assess negative reinforcing and aversive properties of drugs (Schechter and Calcagnetti, 1993). Pairing of contextual information with emesis induced by LiCl can cause CPA in animals (Frisch et al., 1995). Administration of LiCl also results in dose-related CPA (Turenne et al., 1996; Tenk et al., 2005). In this study, we found that activation of PV^+^ interneurons impaired LiCl-induced CPA formation. We proposed that this inhibition might be mediated by the indirect D1^+^ SPN activation. Consistently, our data showed that direct activation of D1^+^ SPNs induced CPP and direct suppression of D1^+^ SPNs induced CPA, suggesting dSPNs are critically involved in both CPP and CPA formation. Our study suggests that activities of PV^+^ interneurons have a critical impact on aversive behaviors by their regulations of accumbal output pathways.

The NAc, an affective nod with the limbic system, is linked to positive and negative emotional valence (Berridge and Kringelbach, 2015). It has been proposed that two populations of NAc outputs exhibit opposing control over valenced behaviors that dSPNs mediate reinforcement and reward, iSPNs are associated with punishment and aversion (Soares-Cunha et al., 2016). In addition, the NAc aids in obtaining motivationally relevant goals, action selection, exploration of novel stimuli, and integrating cognitive and affective information (Floresco, 2015). The regulations of NAc interneurons on emotional valence and other functions are unknown. CPP or avoidance (CPA) is one of the most popular models to study the motivational effects of drugs or other stimuli in experimental animals (Tzschentke, 2007; Sun et al., 2018). When drugs or stimuli bring reward, the animals may show preference for the conditioned place, conversely, when drugs or stimuli bring aversion, the animals show avoidance for the conditioned environments. CPP/A is based on the associative learning that account for the development of an emotional response (Cardinal et al., 2002). After conditioning, the exposure of conditioned place can evoke a representation of affect, such as expectation of reward, which means a pure emotional value tagged to a stimulus is associated with conditioned place. Emotion has some important consequences, including altered motivational behaviors (Pessoa, 2017). So the motivational behaviors (preference or avoidance for the conditioned place) after conditioning might be the result of the emotional value produced by the drug or stimulus. However, the CPP/A models can hardly separate feeling and motivational behaviors. Further studies using other rodent models, such as operant conditioning or self-administration, may reveal the role of accumbal PV^+^ interneurons in regulation of emotion and motivation.

In this study, we found CR^+^ interneurons did not significantly change the emotional states. One possibility is that CR^+^ interneurons provide an equal inhibition to dSPNs and iSPNs. Another possibility is likely due to their low abundance in mouse striatum (Petryszyn et al., 2014). Till now, little is known about the physiological or synaptic functions of accumbal CR^+^ interneurons. Thus, this study provides evidence that PV^+^ and CR^+^ GABAergic interneurons in the NAcc play distinct roles in the regulation of accumbal output pathways and emotional valence. Future studies should identify the functions of all GABAergic interneurons in the NAc to expand our understanding of accumbal local circuitry and the lateral regulations of accumbal output pathways.

## Data Availability

All datasets generated for this study are included in the manuscript.

## Ethics Statement

This study was carried out in accordance with the National Institutes of Health Guide for the Care and Use of Laboratory Animals and were approved by Animal Care and Use Committee of Shanghai Medical College of Fudan University.

## Author Contributions

XL and LM designed the research. XL and XC analyzed the data and wrote the article. XC performed the research. CM and ZL performed the behavioral tests.

## Conflict of Interest Statement

The authors declare that the research was conducted in the absence of any commercial or financial relationships that could be construed as a potential conflict of interest.
